# Hypothermia treatment reduced cyclin-dependent kinase 5-mediated inflammation in ischemic stroke and improved outcomes in ischemic stroke patients

**DOI:** 10.6061/clinics/2020/e1992

**Published:** 2020-07-06

**Authors:** Sombat Muengtaweepongsa, Winchana Srivilaithon

**Affiliations:** I Center of Excellence in Stroke, Faculty of Medicine, Thammasat University, Center of Excellence in StrokeFaculty of MedicineThammasat UniversityThailandThailand.; II Department of Emergency Medicine, Faculty of Medicine, Thammasat University, Thailand.

Dear Editor,

We read with interest the article by Sun et al. concerning the reduction of inflammation with therapeutic hypothermia in acute ischemic stroke (1). In this study, the authors demonstrated the improvement of inflammatory biomarkers after hypothermia treatment in both animal cerebral infarct models and patients with ischemic stroke.

Inflammation is an important step in the ischemic cascade. Therapeutic hypothermia, newly substituted by targeted temperature management (TTM), shows promising effects against the ischemic cascade via multiple mechanisms. In addition to the neuroprotective effects of TTM, their anti-inflammatory mechanism is most essential. TTM has become a standard treatment for cerebral ischemic damage due to cardiac arrest. Unfortunately, many clinical trials failed to demonstrate the benefit of TTM in ischemic stroke; therefore, the routine use of TTM in ischemic stroke is still controversial (2).

Almost all the clinical trials of TTM in ischemic stroke relied on systemic cooling with surface and endovascular methods. These two methods are effective enough to bring down the core temperature to 33°C, which is the target point of the treatment (3). This study relied on the local head cooling by one of the Chinese companies from Harbin. The details of the device were not mentioned in the manuscript. Selective brain cooling with this helmet alone had failed to demonstrate its effectiveness for hypothermic treatment (4).

The temperature measured via the tympanic membrane may be fully reflect the real temperature in the brain. In addition, the tympanic membrane is not a good site for core temperature measurement. The tympanic membrane temperature is lower than the core temperature by 0.5°C (5). The target temperature of 33 to 35°C in the tympanic membrane might not be effective for the hypothermia treatment.

The day when the NIHSS was recorded after the treatment was not mentioned in the manuscript. The NIHSS alone may not be convincing enough for clinical outcome measurement. The modified Rankin scale (mRS) at 3 months after treatment is the most reliable outcome measurement for acute ischemic stroke (6).

This study was a parallel trial in both animals and humans. The authors successfully demonstrated that the effect of neuroprotection due to anti-inflammatory mechanisms is consistent in preclinical and clinical stages. This study has an influence on the development of the future TTM trials in ischemic stroke.
